# Randomized Controlled Trial of Strain-Specific Probiotic Formulation (Renadyl) in Dialysis Patients

**DOI:** 10.1155/2014/568571

**Published:** 2014-07-24

**Authors:** Ranganathan Natarajan, Bohdan Pechenyak, Usha Vyas, Pari Ranganathan, Alan Weinberg, Peter Liang, Mary C. Mallappallil, Allen J. Norin, Eli A. Friedman, Subodh J. Saggi

**Affiliations:** ^1^Kibow Biotech, Inc., 4781 West Chester Pike, Newtown Square, PA 19073, USA; ^2^Mount Sinai School of Medicine, New York, NY 10029, USA; ^3^Downstate Medical Center, State University of NY, New York, NY 11203, USA

## Abstract

*Background*. Primary goal of this randomized, double-blind, placebo-controlled crossover study of Renadyl in end-stage renal disease patients was to assess the safety and efficacy of Renadyl measured through improvement in quality of life or reduction in levels of known uremic toxins. Secondary goal was to investigate the effects on several biomarkers of inflammation and oxidative stress. *Methods*. Two 2-month treatment periods separated by 2-month washout and crossover, with physical examinations, venous blood testing, and quality of life questionnaires completed at each visit. Data were analyzed with SAS V9.2. *Results*. 22 subjects (79%) completed the study. Observed trends were as follows (none reaching statistical significance): decline in WBC count (−0.51 × 10^9^/L, *P* = 0.057) and reductions in levels of C-reactive protein (−8.61 mg/L, *P* = 0.071) and total indoxyl glucuronide (−0.11 mg%, *P* = 0.058). No statistically significant changes were observed in other uremic toxin levels or measures of QOL. *Conclusions*. Renadyl appeared to be safe to administer to ESRD patients on hemodialysis. Stability in QOL assessment is an encouraging result for a patient cohort in such advanced stage of kidney disease. Efficacy could not be confirmed definitively, primarily due to small sample size and low statistical power—further studies are warranted.

## 1. Introduction

During coevolution with microbes, the human intestinal tract has been colonized by thousands of bacterial species [1, 2]. Gut-borne microbes outnumber the human body cells by a factor of ten [3]. Recent metagenomic analysis of human gut microbiota has revealed the presence of 3.3 million genes, compared to mere 23 thousand known human genes [4–6]. Microbial communities perform the majority of biochemical activities on the planet and play integral roles in human metabolism and immune homeostasis [7]. Recently, evidence of benefits for human health from intestinal microbiota and probiotic microbes has expanded rapidly [8–12].

Probiotics, “live microorganisms which when administered in adequate amounts confer a health benefit on the host [13],” are predominantly found in fermented dairy foods (yogurt, kefir, and cheese). Although the expansion of awareness and use of probiotics has raced ahead of the scientific knowledge of mechanisms by which they impact health, probiotics appear with increasing frequency in various foods, beverages, and supplements and are increasingly utilized in clinical settings. As their safety and health benefits are established, it is reasonable to anticipate that they will be incorporated into a growing number of clinical regimens, either independently or as adjunct/combined treatments.

General awareness of the rising global prevalence of kidney disease has been steadily growing among medical and public health professionals [14–16]. Kidney disease is the eighth leading cause of death in the U.S. [17], with approximately 600.000 patients in end-stage renal disease (ESRD, most receiving dialysis) and over 20 million in earlier stages of chronic kidney disease (CKD) [18]. As the population continues to age and the epidemiological shift from acute infectious to chronic metabolic diseases progresses, contributing factors to kidney disease (obesity, diabetes, and hypertension) become epidemic. Kidney disease may turn into a major health crisis in the USA and globally. The use of dietary supplements is a promising approach and should be included in any strategy to reduce the likelihood of such crisis.

**Figure 1:**
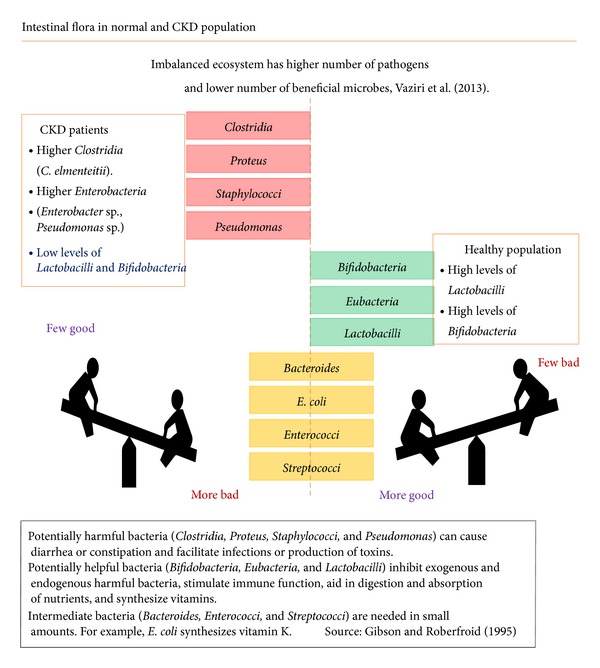
Dysbiosis in CKD.

The role of digestive [19] and immune [20] systems, as well as inflammatory [21] and oxidative stress [22, 23] functions, in the progression of kidney disease has been emphasized by researchers in the past decade. Current data have highlighted an integrated and perhaps a causal relationship between the observed clinical outcomes and the role of an activated immune system in uremia. (Please see Figure 1 for elucidation of dysbiosis.)

The potential utilization of oral sorbents and probiotics has been continuously explored as a complementary strategy for CKD over the past 15 years. Initial* in vitro* R&D lab studies were performed including the use of a simulated human intestinal microbial ecosystem (SHIME), a five-step biochemical reactor to mimic stomach, small intestine, and ascending, transverse, and descending colonic environments [24]. Further exploratory studies of orally administered probiotic bacteria were performed in 5/6th nephrectomized rats [25] and mini pigs [26], in cats [27] and dogs with kidney failure, and in humans [28, 29] with CKD and ESRD [30]. (Two unpublished studies by veterinary doctors: Carol L. Galka, DVM, Companion Animal Care Center, Caro, MI (*n* = 2), and Gary van Engelenberg, DVM, CVA, Iowa Veterinary Acupuncture Clinic, Des Moines, IA (*n* = 6).)

To determine whether daily probiotic bacterial treatment improves or delays the onset of CKD signs and symptoms, several pilot-scale human clinical trials were conducted. They showed that a proprietary probiotic formulation can utilize various nitrogenous uremic toxins as nutrients for growth of beneficial gut microbes. Specifically formulated probiotic microbial strains keep uremic toxins from accumulating to highly toxic levels. In December 2012, two most recent studies were completed: an open label, observational dose escalation study in CKD stages 3 and 4 patients at Thomas Jefferson University (Philadelphia, PA) [31] and the current study. The former study aimed to confirm the safety and tolerability of several doses of the formulation as well as to quantify the improvements in quality of life (QOL) and to explore several molecular biomarkers. The primary goal of the current study was to confirm the efficacy of the formulation in effecting a measurable quality of life improvement and reducing the levels of commonly known uremic toxins. The secondary goal was to investigate the product's effects on some inflammation and oxidative stress biomarkers.

## 2. Subjects and Methods

### 2.1. Study Design

**Figure 2:**
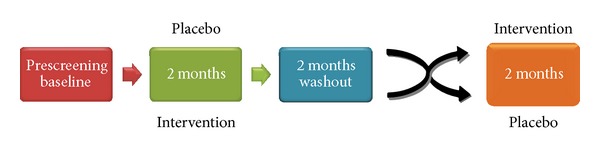
Study design.

A 6-month randomized, double-blind, placebo-controlled crossover study of an orally administered, strain-specific probiotic formulation (Renadyl, Kibow Biotech, Inc., Newtown Square, PA) in ESRD patients receiving dialysis treatment was initiated at the Downstate Medical Center (DMC, Brooklyn, NY) in April 2011 (Figure 2). The study protocol had been approved by the DMC Institutional Review Board (NIH registry #NCT01450709), and written informed consent was obtained from each participant at enrollment. The study participants enrolled voluntarily were prequalified and selected based on prior medical history and the inclusion/exclusion criteria.

*Primary endpoints* were defined as measurable improvement in the quality of life (in accordance with modified SF36 questionnaire) and in the levels of biochemical markers, such as urea and creatinine, hematological values (CBC), and hepatological function.* Secondary endpoints* included the measurements of several biomarkers of inflammation and oxidative stress (indoxyl metabolites, p-cresyl sulfate, serum pentosidine, *β*-2 microglobulin, NF-*κ*B, and sCD30).

During the screening (T0), baseline values were obtained, and each patient was examined, randomly assigned to either treatment or control group, and initiated on a dose of 2 capsules thrice daily with meals (Table 1). Each capsule contained either the probiotic formulation—30 billion CFU of* S. thermophilus* KB 19,* L. acidophilus* KB 27, and* B. longum* KB 31—or placebo, which consisted of a 1 : 1 blend of cream-of-wheat and psyllium husk (both formulation and placebo manufactured by ADH, Congers, NY). The second visit was scheduled at the end of month 2 (T1), at which point the first treatment period ended and the 2-month washout period began. At month 4, the washout period ended and second treatment period began. The final follow-up visit occurred at month 6 (T2), the study end. Participants underwent routine physical examinations and blood draws, completed modified SF-36 QOL questionnaires, and were monitored for compliance with the study protocol at each visit. (Exception: at month 4, patients visited to obtain the product, with no exams/measures.)

### 2.2. Inclusion and Exclusion Criteria

The* inclusion criteria *defined the potential participant population as those aged 18–80 and diagnosed with CKD stage V (ESRD, currently receiving hemodialysis treatment).

The* exclusion criteria* limited the study population by excluding (1) pregnant or nursing women, (2) those with HIV/AIDs or liver disease diagnoses, (3) those with active dependency on controlled substances and alcohol, (4) those on anticoagulant therapy regimen, (5) those refusing to sign the informed consent form, and (6) those with social conditions or medical debilitating disease/disorder, which, in the judgment of the investigator, would interfere with or serve as a contraindication to adherence to the study protocol or ability to give informed consent or affect overall prognosis of the patient.

### 2.3. Laboratory Methods

#### 2.3.1. Biochemistry and Hematology

No changes in the dialysis prescription of these patients occurred during the study period. Complete blood counts and serum biochemical testing were performed at each patient's dialysis treatment facility at DMC, either Parkside (PS, patients 1–12, 20, 25–28) or Kings County (KC, 13–19, 21–24). Glucose was monitored closely, if the patients were diabetic.

#### 2.3.2. Uremic Toxins and Inflammation Markers

The secondary aim of the study was to investigate possible changes in markers of inflammation, known to increase in uremia, such as C-reactive protein and NF-*κ*B, as well as such uremic toxins as total and free indoxyl sulfate, total and free indoxyl glucuronide, total and free indole acetic acid (IAA), total and free p-cresyl sulfate, total and free hippuric acid, pentosidine sulfate, *β*-2 microglobulin, 3-carboxyl-4-methyl-5-propyl-2-furan-propanoic acid (CMPF), and uric acid.

Chemicals were measured by HPLC and ELISA. Peripheral blood mononuclear cells (PBMC) were extracted from whole patient blood samples, using Ficoll-Hypaque to form the density gradient, and centrifuged. NF-*κ*B levels were assayed using the TransAM p65 ELISA kit (Active Motif, Carlsbad, CA). Viability of cells was assessed using trypan blue exclusion. An aliquot of the cells extracted was used for lysis. The nuclear content from the aliquot was extracted using the protocol from the kit. The final solution was diluted to 12,500 cells/*μ*L using the cell lysis buffer in combination with the protease inhibitor cocktail. The cell extracts were stored at −80°C. Analysis was performed according to the kit instructions.

Serum pentosidine and *β*-2 microglobulin were analyzed using ELISA kits (Novateinbio, cat. no. NB-E10646, and R & D Systems, cat. no. DBM200, resp.). Other chemicals were quantified by HPLC on a Waters Alliance 2695 (Waters, Zellik, Belgium) and two detectors in series (Waters 996 photodiode array detector (PDA) and a Waters 2475 fluorescence detector (FLD)), using methods of Taki and Niwa [32] and Martinez et al. [33].

To determine the total serum concentration, 75 *μ*L of sample was diluted with 195 *μ*L of HPLC water, followed by heating at 95°C for 30 min. Then the samples were placed on ice for 10 minutes and subsequently passed through a molecular filter (Amicon Ultra 0.5 mL) with a 30.000 Da cut-off weight. To measure the free fraction, untreated serum samples were filtered prior to heating. In order to correct for system performance variations, 25 *μ*L of fluorescein (50 mg/L) was added to 225 *μ*L of ultrafiltrate as internal standard. Subsequently, this was transferred to an autosampler vial and 50 *μ*L thereof was injected in the column.

The separation was performed at room temperature on a reversed-phase XBridge C8 column (3.5 *μ*m, 150 mm × 4.6 mm, Waters) with an Ultrasphere ODS guard column (5 *μ*m, 5 mm × 4.6 mm, Beckman Instruments). The mobile phase consisted of a 50 mM ammonium formate buffer (mobile phase A, pH 3.0) and methanol (mobile phase B). A gradient elution at a flow of 1 mL/min was performed with an initial composition of 100% phase A and held at this composition for 3 min. Then, this increased to 100% B in 31 min and this composition was held for 3 min and finally a reequilibration was done. For uric acid, hippuric acid and CMPF chromatograms were extracted from the PDA data at 300 nm, 245 nm, and 254 nm, respectively. Fluorescence excitation and emission wavelengths were optimized for the other compounds: *λ*_ex_ = 272 nm and *λ*_em_ = 374 nm for indoxyl sulfate and indoxyl glucuronide, *λ*_ex_ = 264 nm and *λ*_em_ = 290 nm for p-cresyl sulfate and p-cresyl glucuronide, *λ*_ex_ = 272 nm and *λ*_em_ = 340 nm for indole acetic acid, and *λ*_ex_ = 443 nm and *λ*_em_ = 512 nm for the internal standard. Five point calibration curves were generated. Good linearity was observed for all compounds. For the regression calculation a weighing factor of 1/*x* was used for all data points.

After initial analysis, to link some of the results obtained to the markers of inflammation, a sCD30 biomarker of T-cell activation was investigated. This marker has previously been shown to be elevated in patients with CKD [34]. Also, lower levels of sCD30 have been associated with better prognosis in kidney transplant patients [35]. The levels of sCD30 were measured by ELISA kit (eBioscience, San Diego, CA, cat. no. BMS240).

### 2.4. Statistical Methods

All variables were analyzed for change with reference to the values obtained during the placebo study period. All measures were modeled via the PROC MIXED procedure in SAS, similar to an analysis of variance for repeated measures. Due to the fact that repeated measurements within each patient may be correlated, the Mixed Model procedure allows one to model this “correlation structure,” commonly referred to as a covariance pattern. This accurate estimate will allow for improved estimates of the standard errors of measurement and therefore more powerful tests.

There are a number of various covariance structures to choose from. Three of the more common covariance structures include “compound symmetry” (CS), for correlations that are constant for any two points in time, “autoregressive order one” (AR1), for correlations that are smaller for time points further apart, and “unstructured” (UN), which has no mathematical pattern within the covariance matrix. Other covariance structures that are usually tested include the Toplitz (TOEP) and the heterogeneous compound symmetry structure (CSH).

A likelihood ratio test or a procedure known as Akaike's information criterion (AIC) is used to discern which covariance pattern allows for the best fit [36]. Therefore the “compound symmetry” (CS) structure was chosen. Adjusted means at each time point were then generated with adjusted standard errors. *P* values were not adjusted for multiple comparisons and the inflation of the Type I error.

SAS system software V 9.2 (SAS Institute Inc., Cary, NC) was used for all statistical analyses.

### 2.5. Patient Adherence

Patient compliance and adherence was assessed by pill count and stool culture to verify probiotic growth during study and absence during placebo period. Fecal samples were analyzed at Kibow's lab for the presence of the three strains comprising the study formulation using microbiological methods of plating, enumeration, and counting the colonies on appropriate and specific growth media on agar plates.

## 3. Results

### 3.1. Patient Baseline Demographics and Epidemiology

Among the 22 participants, the average age was 54 (range 29–79) and the predominant sex was female (*n* = 16, 73%). Vital sign values were as follows: systolic blood pressure (BP) averaged at 148 mmHg (range 100–188 mmHg), diastolic BP—76 mmHg (53–111 mmHg), respiration—17/min (16–18), and pulse—76/min (55–96/min). All medications, prescribed and administered to each patient prior to the initiation of the study and the Renadyl regimen, were either continued without change or reassessed and substituted by an alternative therapeutic modality, in accordance with the accepted standards of care.

### 3.2. Study Results

Of 28 participants, 22 (79%) completed three visits. Two patients withdrew consent after the baseline visit (T0), one of them due to nausea and vomiting. Both of these patients were on placebo. The capsules administered were vegetarian gel caps size 0 at a dosage level of two capsules three times a day. 4 more dropped out after visit 1 (T1): 1 was transferred to a different facility, 2 withdrew consent, and 1 passed away of unrelated causes (see Section 3.3).

Administration of probiotics was accompanied by the following trends (not reaching statistical significance; see Tables 2, 3, and 4): decline in WBC count (change of −0.51 × 10^9^/L, *P* < 0.057) and reductions in the levels of total indoxyl glucuronide (−0.11 mg%, *P* < 0.058) and C-reactive protein (−8.62 mg/L, *P* < 0.071). No statistically significant changes were observed in the levels of other uremic markers or measures of QOL.

No major issues were encountered with regard to patient adherence to the treatment regimen. Average adherence amounted to 92.5%, with a standard deviation of 13.7%.

### 3.3. Adverse Events

The study was monitored according to the best clinical practices as per the nephrology institutional clinical standards of Downstate Medical Center, State University of New York, Brooklyn, NY. There was one Severe Adverse Event with a lethal result, unrelated to the study protocol—myocardial infarction while sleeping at home (underlying atherosclerotic and coronary heart disease). Patient issues included a long-term smoking history at a rate of several packs per day, continued strenuous employment despite multiple health conditions, 6 years of dialysis treatment comorbid with severe hyperparathyroidism and hyperphosphatemia, accompanied by poor adherence to and compliance with dialysis treatments, medications, diet, and phosphate binder regimen, as well as poor to no follow-up with specialists. Five other patients withdrew consent, 1 due to nausea and vomiting, 1 because of being transferred to a different facility in the state of Maryland, and the other 3 for unspecified reasons. Also, there was another patient who withdrew consent, complaining of nausea and vomiting, but later reaffirmed consent.

## 4. Discussion

Toxicity from the accumulation of uremic toxins is a concern for kidney disease patients. Concentrations of uremic solutes increase as the disease progresses from CKD to ESRD [37]. The European Toxin workgroup (EUTOX) has classified many uremic toxins based on their molecular weights and their protein binding property [38]. Though urea is generally nontoxic, it can degrade to highly toxic cyanate, which binds to proteins by carbamylation and modifies them, including serum albumin. Recent study by Berg et al. [39] showed that carbamylated serum albumin is a risk factor for mortality in patients with kidney failure. As early as 1998, it was shown that CKD patients face higher risk of cardiovascular (CV) problems, with CV mortality 10–20 times higher than in the general population [40]. Therefore, it may be necessary to reduce CKD patients' urea levels either with medication or through interventions like probiotic supplementation (some lactic acid bacteria can metabolize urea).

Probiotics have been reported to enhance intestinal health for centuries [41]. Scientific proof has now been obtained that confirms their positive effects on human health in general [42]. The application of probiotics in various diseases has intensified, as extensive research efforts help understand how they shape human health and how their composition changes in diseased states [43]. The application of probiotics in ESRD management has been investigated in both experimental and clinical settings [44]. Recently, deeper insight was gained into probiotics' positive effects on kidney disease progression—possible mechanisms include anti-inflammatory (addressing imbalances of gut dysbiosis) and antioxidant (addressing deficiencies in free radical signaling—generation of reactive oxygen species in the gut) routes [45].

### 4.1. Probiotics and Renal Health

It has been demonstrated previously that gut microflora can affect the concentrations of uremic toxins in animals. Prakash and Chang were able to continuously reduce blood urea nitrogen in azotemic rats by oral administration of microencapsulated genetically engineered live cells containing living urease-producing* E. coli* DH5 [46]. Based on this concept, Ranganathan et al. carried out rat studies using 5/6th nephrectomized animals fed with a probiotic cocktail of* Lactobacilli*,* Bifidobacteria*, and* S. thermophilus* [25]. Results showed a significantly prolonged life span for the uremic rats, in addition to reduced blood urea-nitrogen (BUN) levels. Studies were subsequently carried out in 5/6th nephrectomized Gottingen mini pigs [26]. Here, also there was a reduction in BUN and creatinine levels, indicating that the probiotic supplementation prevented the accumulation of these toxins in the blood. These results were further evaluated clinically by Palmquist in feline azotemia [27]. Studies in 7 cats showed statistically reduced levels in BUN and creatinine levels and demonstrated significantly improved quality of life (QOL). The product is currently marketed worldwide for cats and dogs with moderate-to-severe kidney failure (Azodyl, Vetoquinol SA, http://www.vetoquinol.com/).

In human studies, Simenhoff et al. demonstrated that hemodialysis patients who were fed* L. acidophilus* NCFM had significantly lower blood dimethylamine and nitrodimethylamine levels [47, 48]. Simenhoff was the first researcher to demonstrate the growth of pathogenic bacteria which is referred to as “small bowel bacterial overgrowth” (SBBO). The NCFM strain is well known, and the genome has been sequenced by Todd Klaenhammer's group [49]. Subsequent to the success of the formulation for cats and dogs described above, a similar formulation for humans was evaluated clinically in a 6-month randomized, double-blind, placebo-controlled, crossover trial in CKD stages III and IV patients in four countries [28, 29]. Forty-six patients were studied in this trial. BUN levels decreased in 29 patients (*P* < 0.05), creatinine levels decreased in 20 patients (no statistical significance), and uric acid levels decreased in 15 patients (no statistical significance). Almost all subjects reported having experienced a substantial perceived improvement in their quality of life (*P* < 0.05). This product is also currently marketed to CKD patients (Renadyl, Kibow Biotech, Inc., Newtown Square, PA, USA, http://www.renadyl.com/).

Previous multicenter trials in cohorts of CKD stages 3-4 patients showed that concentrations of uremic toxins (urea, uric acid, and creatinine) were reduced when study subjects were treated with the study formulation at 90 billion CFU/day dosage [29]. Open label, dose escalation observational study in CKD stages 3-4 patients showed statistically significant reductions in creatinine and C-reactive protein, significant improvements in hemoglobin, hematocrit, and physical functioning (QOL measure), trends toward reduction in BUN, potassium, and pain (QOL), and no significant change in mental, emotional, and social well-being [31].

The current study was conducted to assess the safety and efficacy of the formulation in ESRD patients receiving dialysis treatment. The results indicate that the administration of the formulation in ESRD patients is safe and might even have a slight protective effect, as indicated by a trend toward reducing inflammation markers. Since NF-*κ*B pathway is neither activated (important in cases of active infections) nor modulated/suppressed, the formulation appears not to harm immune function. Levels of sCD30 are not affected by the administration either, further confirming that patients are not immunologically compromised by probiotic treatment. Further investigation in a larger population, at a higher dose and over a longer term, might yield mechanistic insights into the probiotic effects on the inflammatory cascade of uremia and the modulation of T-cells in ESRD. The next clinical trial bearing this in mind is underway where hemodialysis and peritoneal dialysis patients will receive 180B CFU/day for a period of 6 months to get better statistical data.

Studies by Vaziri et al. [50] have shown that renal failure patients have an imbalanced gut microflora, while a recent review of the studies with pro- and prebiotics summarized the role of the gut microflora in uremia and CKD [51]. As the review states, it is not well recognized that an important contributing factor to the toxic load leading to CKD originates in the gut. The microbiota that colonize the gut perform such functions as regulating the normal development and function of the mucosal barriers; assisting with maturation of immunological tissues, which in turn promotes immunological tolerance to antigens from foods, the environment, or potentially pathogenic organisms; controlling nutrient uptake and metabolism; and preventing propagation of pathogenic microorganisms. The review concludes that probiotics and prebiotics are very likely to play a therapeutic role in maintaining a metabolically balanced gut and reducing progression of CKD and associated uremia.

In addition, recent studies indicate that such metabolites as phenols and indoles, which are also uremic toxins, come from colonic fermentation [52]. In CKD, protein digestion is impaired; undigested proteins enter the large intestine and are fermented by pathogenic bacteria, eventually forming indoles and phenols, which are then converted to indoxyl and p-cresyl sulfates, glucuronides, and other metabolites.

This study investigated whether probiotic supplementation could lower the concentrations of these putrefactants. For example, the generation rate of indoles, produced from amino acid tryptophan, may be altered by probiotics. As indicated, the values of most biomarkers varied widely and did not reach statistical significance (data omitted), the only exception being a trend toward reduction in the levels of total indoxyl glucuronide. QOL results, likewise, did not show any significance (data omitted), though stability and lack of deterioration in itself are encouraging, given the advanced stage of renal failure.

### 4.2. Study Limitations

The most significant limitation was sample size, affecting the statistical power of the study results. Since this was a pilot trial to establish safety and efficacy, minimal, limited number of patients were chosen. Future larger trials based on the findings of this ESRD and an earlier CKD probiotic trial [32] should be sufficiently powered.

The likeliest explanation of the lack of statistically significant results is that (a) ESRD is an advanced stage of CKD, patients have multiple complications, and the extent of disease is already life-threatening enough to qualify patients for life-sustaining dialysis treatments; (b) dialysis per se does reduce/remove some of the smaller water soluble molecules and uremic toxins like urea; (c) the study was at a dosage of 180B CFU/day for just two months. Despite the short administration of the probiotic one of the uremic toxins indoxyl glucuronide levels showed a decrease. This toxin is generated by gut dysbiosis and cannot be removed by dialysis; hence, reduction in the levels of this toxin indicates a positive response attributed to the probiotic bacteria present in Renadyl. In most cases, the best results to be expected from probiotic supplementation are stabilization of uremic toxin levels and stabilization or improvement of the quality of life. Whether more significant effects are possible—for example, reduction in duration or even frequency of dialysis sessions—remains to be determined from future studies employing larger patient samples.

## 5. Conclusions

Administration of Renadyl in ESRD patients at the dose of 180 billion CFUs per day appears safe and well tolerated. Trends were noted in WBC count, C-reactive protein, and total indoxyl glucuronide, none reaching statistical significance. Other uremic toxins, markers of inflammation and oxidative stress, and quality of life measures did not show statistically significant changes. For more definitive results, especially to confirm the trends observed, a study with a larger sample size is warranted.

## Figures and Tables

**Table 1:** Randomization and blinding (Tx: treatment; PL: placebo).

Patient number.	Period 1	Period 2
1	**Tx**	PL
2	**Tx**	PL
3	**Tx**	PL
5	**Tx**	PL
6	PL	**Tx**
7	PL	**Tx**
8	**Tx**	PL
11	PL	**Tx**
12	PL	**Tx**
13	**Tx**	PL
14	**Tx**	PL
15	PL	**Tx**
16	PL	**Tx**
17	**Tx**	PL
18	**Tx**	PL
19	PL	**Tx**
20	PL	**Tx**
21	**Tx**	PL
25	**Tx**	PL
26	PL	**Tx**
27	**Tx**	PL
28	PL	**Tx**

**Table 2:** Means.

Variable	Tx period	*N*	Mean	Std. Dev.	Median	Min	Max
White blood cells (WBC)	Base	22	6.36	1.33	6.33	3.92	9.45
	Placebo (PL)	21	6.07	1.55	5.48	3.78	10.07
	Treatment (Tx)	21	5.57	1.17	5.75	3.46	8.04

C-reactive protein (CRP)	Base	21	8.89	9.65	5.00	0.30	40.00
	PL	18	11.28	19.36	5.31	0.51	85.00
	Tx	19	5.10	3.80	4.00	0.30	14.00

Total indoxyl glucuronide (TIG)	Base	22	0.75	0.23	0.70	0.37	1.31
	PL	22	0.75	0.25	0.73	0.33	1.30
	Tx	22	0.67	0.21	0.67	0.30	1.19

**Table 3:** Least squares means.

Variable	Estimate	Std. error	*t* value	Pr > |*t*|	Alpha	Lower	Upper
WBC	6.0157	0.2981	20.18	<0.0001	0.05	5.3894	6.6419
	5.5099	0.2965	18.58	<0.0001	0.05	4.8868	6.1329

CRP	13.7221	3.2992	4.16	0.0011	0.05	6.5946	20.8495
	5.1068	3.0324	1.68	0.1160	0.05	−1.4444	11.6580

TIG	0.7617	0.04643	16.41	<0.0001	0.05	0.6649	0.8586
	0.6536	0.04551	14.36	<0.0001	0.05	0.5586	0.7485

**Table 4:** Differences of least squares means.

Variable	Tx period	Estimate	Std. error	*t* value	Pr > |*t*|	Lower	Upper
WBC	PL-Tx	0.5058	0.2486	2.03	**0.0569**	−0.0164	1.028
CRP	PL-Tx	8.6153	4.3757	1.97	**0.0707**	−0.8379	18.0685
TIG	PL-Tx	0.1081	0.05377	2.01	**0.0579**	−0.00401	0.2203
